# The Hepatitis C Cascade of Care among HIV Infected Patients: A Call to Address Ongoing Barriers to Care

**DOI:** 10.1371/journal.pone.0102883

**Published:** 2014-07-18

**Authors:** Edward R. Cachay, Lucas Hill, David Wyles, Bradford Colwell, Craig Ballard, Francesca Torriani, William C. Mathews

**Affiliations:** 1 Department of Medicine, Owen Clinic, University of California San Diego, San Diego, California, United States of America; 2 Department of Medicine, Division of Infectious Diseases, University of California San Diego, San Diego, California, United States of America; 3 Skaggs School of Pharmacy and Pharmaceutical Sciences, University of California San Diego, San Diego, California, United States of America; University of Pisa, Italy

## Abstract

**Background:**

The aims were to investigate the hepatitis C (HCV) cascade of care among HIV-infected patients and to identify reasons for not referring for and not initiating HCV therapy after completion of HCV treatment staging.

**Design and Methods:**

Retrospective cohort analysis of HIV-infected patients under care at the University of California, San Diego (UCSD). We identified patients screened for and diagnosed with active HCV infection. Logistic regression analyses were used to identify factors associated with lack of referral for HCV therapy. Electronic medical records were reviewed to ascertain reasons for not initiating HCV therapy.

**Results:**

Between 2008 and 2012, 4725 HIV-infected patients received care at the UCSD Owen clinic. Most patients [4534 (96%)] were screened for HCV, 748 (16%) patients had reactive serum HCV antibodies but only 542 patients had active HCV infection. Lack of engagement in care was the most important predictor of non-referral for HCV therapy [odds ratio (OR): 5.08, 95% confidence interval 3.24–6.97, p<0.00001]. Other significant predictors included unstable housing (OR: 2.26), AIDS (OR: 1.83), having a detectable HIV viral load (OR: 1.98) and being non-white (OR: 1.67). The most common reason (40%) for not initiating or deferring HCV therapy was the presence of ongoing barriers to care.

**Conclusions:**

Screening for HCV in HIV-infected patients linked to care is high but almost half of patients diagnosed with HCV are not referred for HCV therapy. Despite improvements in HCV therapy the benefits will not be realized unless effective measures for dealing with barriers to care are implemented.

## Introduction

Approximately 25% of persons living with human immunodeficiency virus (HIV) infection are coinfected with hepatitis C (HCV) [1]. Guidelines recommend screening for HCV in persons infected with HIV upon establishment of care [2]. For example, in a large diverse cohort of HIV clinics across the United States at the end of 2011, 85% of HIV-infected persons received HCV antibody screening within 3-months of enrolling in care [3]. Despite the high prevalence of HCV among HIV-infected persons and major advances in HCV therapy, access to HCV treatment remains low in this population across the United States and Europe [4], [5]. HCV has become the leading cause of liver-related morbidity and mortality in developed countries, and has contributed to increased health care costs [6],[7]. Thus, there is an urgent need to increase both access to treatment and treatment uptake of HCV in HIV-infected persons.

With the advent of direct acting antiviral agents for treatment of HCV there is an opportunity to cure HCV in most coinfected persons [8]; however, little is known about factors influencing HCV treatment referral and disposition following HCV diagnosis and establishing HIV care [9]. Understanding the reasons for non-referral for HCV treatment and for not initiating HCV therapy among those HIV-infected patients referred for HCV therapy may help us to develop targeted programs to narrow gaps in access to HCV therapy [10]. The present study was conducted to identify factors associated with non-referral for HCV treatment consideration, and to characterize reasons for not starting HCV therapy after completion of HCV treatment staging. The study also describes the HCV cascade of care among patients with known HCV infection that received care at the Owen Clinic, University of California at San Diego (UCSD).

## Methods

### Patients and HCV model of care

We conducted a retrospective cohort study of HIV-infected persons with active HCV infection under care at UCSD Owen Clinic. Active HCV infection was defined as having both a reactive HCV antibody and detectable HCV RNA (if available), or a non-reactive HCV antibody and detectable HCV RNA. In 2008, a multidisciplinary HCV coinfection primary care-based program was implemented at UCSD, with an inclusive protocol aimed at increasing HCV treatment uptake among HIV co-infected patients, including those with ongoing drug and/or alcohol abuse and neuropsychiatric disease [11]. The HCV coinfection clinic operates as one clinic session per week and is co-located within the UCSD Owen HIV Clinic. Owen Clinic is funded by The Health Resources and Services Administration (HRSA) through the Ryan White C.A.R.E. Act Part C Early Intervention Services (EIS) Grant Program. Since our coinfection clinic is embedded within our HIV primary care clinic, the only requirement to initiate the referral process is that HIV primary providers place a HCV referral in our electronic medical record (EMR). The EMR referral documentation served to identify HCV referral status. This study was conducted according to the principles expressed in the Declaration of Helsinki. The study was approved by the UCSD Human Research Protection Program, project# 071931. Patient records/information was anonymized and de-identified prior to analysis.

### Study design

HIV/HCV coinfected patients were categorized based on our EMR HCV treatment referral status into two groups, referred or non-referred. To investigate reasons for HCV non-referral, we compared the referred vs. non-referred groups with respect to six major clinical domains. The six clinical domains included demographic characteristics, HIV laboratory markers (CD4 and viral load), history of advanced liver disease, presence of ongoing barriers to care, level of engagement in care, and presence of concurrent medical comorbidities that could contraindicate the use of interferon-based therapy. Next, using EMR HCV referred status, we identified the number of HIV-infected persons referred for HCV therapy who attended at least one HCV coinfection clinic visit, and of them, the proportion of patients who completed clinical staging for HCV therapy. To identify reasons for not initiating HCV therapy, we used as a denominator the number of patients who completed HCV staging and did not initiate HCV therapy.

### Study domain definitions

*Advanced liver disease* was defined as having a prior episode of liver decompensation (hepatic encephalopathy, ascites, spontaneous bacterial peritonitis, bleeding varices or portal gastropathy), a Childs-Pugh score ≥ B7, or the presence of severe portal hypertension with esophageal varices.

*Barriers to care* were defined as the presence of any of the following 3 categories as documented in the clinical notes of our electronic medical records: (1) self-reported ongoing illicit drug or alcohol use within 3 months of last attended HIV clinical visit or first HCV attended clinic visit whichever occurred last; or loss to follow-up attributed to alcohol or drug use; (2) psychiatrist or primary care provider documentation of ongoing uncontrolled neuropsychiatric disease; and, (3) poorly controlled HIV disease [CD4 ≤200 cells/mm^3^ and detectable HIV viral load (>40 copies/dl)] in the context of alcohol or drug use.

*Engagement in care* was defined as having 2 or more visits with a primary care physician in our system separated by 3 or more months in each calendar year for the entire defined study period or until date of referral for HCV therapy, unless patient transferred care or was incarcerated. Clinic visits to urgent care or UCSD emergency department were excluded.

Four main categories of *concurrent medical comorbidities* were accounted for: (1) cardiovascular disease, including history of myocardial infarction within the last 6 months, congestive heart failure stage 2 or higher according to the New York Heart Association functional classification system, arrhythmia that required an implantable device; (2) chronic kidney disease stage 3b or higher (glomerular filtration rate <45 mL/min/1.73 m^2^); (3) active diagnosis of malignancy other than hepatocellular carcinoma or basal cell cancer of skin; and (4) known neurologic, dermatologic, pulmonary, hematologic and metabolic conditions where interferon plus ribavirin-based therapy could be contraindicated (Table S1 in File S1).

Data was collected by identification of each patient's diagnostic problem list and with manual verification of each clinic visit for every patient during the study period. A random sample of 35% of electronic medical records was independently reviewed by a different investigator (E.C.) to assure agreement and validity of data collection, this review focused on fidelity of ascertainment of ongoing barriers to care definitions as the rest of study domains were abstracted from our EMR.

### Statistical analysis

We used chi-square analysis or Wilcoxon-rank sum test to compare categorical or continuous variables respectively. Bivariate analyses were conducted to investigate factors associated with lack of referral for HCV therapy. Logistic regression analyses were conducted to adjust for significant factors identified in bivariate analyses, using NCSS version 8.0 (Kaysville, Utah, USA).

## Results

Between 1 January 2008 and 31 December 2012, 4725 HIV-infected patients had at least one primary care visit care at the UCSD Owen Clinic. Most patients were screened for HCV [4534 (96%)] and 748 (16%) had reactive serum antibodies against HCV. There were 303 and 445 patients in the HCV referred and non-referred groups, respectively. However, 186 patients in the non-referred group had valid reasons for HCV non-referral such as spontaneous HCV clearance (n = 138), HCV cure with interferon prior to 2008 (n = 34), and equivocal HCV antibody with serial undetectable repeated HCV viral loads (n = 14), and were excluded from the analysis. Thus, 562 HIV-infected patients had active HCV infection in need of HCV therapy according to current guidelines [12]. Most HIV-infected patients with active HCV infection (84%) were male with a median (range) age and CD4 cell count of 48 years (19–75) and 388 cells/mm^3^ (5-1777). By race/ethnicity 33% were non-white, 20% Hispanic and 60% had undetectable HIV viral load (<40 copies/ml). The routes of HIV acquisition included 30% men who have sex with men (MSM), 21% MSM with intravenous drug use (IDU), 15% Men IDU unwilling to disclose sexual orientation, 14% heterosexual with IDU, 8% heterosexual not IDU, 5% women IDU unwilling to disclose sexual risk behavior, 3% transfusion (hemophilia) and 4% unknown.

In comparison to the HCV referred group, the non- referred group included a significantly higher proportion of non-white patients, MSM without IDU history, and uncontrolled HIV infection manifested by lower CD4 cell counts and higher proportion of detectable HIV viral load (Table 1). The HCV non-referred group also had a higher proportion of individuals not engaged in HIV care and with unstable housing than patients in the referred group. Unexpectedly, the non- referred group included a lower proportion of patients with ongoing neuropsychiatric disorders and alcohol/drug abuse; however, the prevalence of ongoing neuropsychiatric disorders and drug/alcohol abuse was nonetheless substantial among patients referred for HCV therapy, 47% and 39%, in the HCV referred group, respectively. There were no differences between the two groups in terms of age, gender distribution, history of advanced liver disease, and medical comorbidities that may preclude interferon-based therapy initiation (Table 2). Description of common medical comorbidities that could contraindicate the use of interferon and ribavirin or took medical priority over HCV referral is presented in Table S2 in File S1.

**Table 1:** Demographic characteristics, CD4 cell count and HIV viral load of patients' referred and non-referred for hepatitis C treatment consideration.

Clinic characteristic	Referred (n = 303)	Non-referred (n = 259)	*P* value
Median age − years (range)	48 (19–75)	49 (20–71)	0.30
Sex: Male (%)	256 (85)	215 (83)	0.64
Race: Non- White (%)	84 (27)	101 (39)	**0.001**
Ethnicity: Hispanic (%)	50 (17)	55 (21)	0.15
HIV risk factors			**<0.0001**
MSM/bisexual (%)	76 (25)	90 (35)	
Heterosexual (%)	14 (5)	32 (12)	
Intravenous drug use (%)	78 (26)	30 (12)	
Hemophilia (%)	9 (3)	8 (3)	
MSM and intravenous drug use	69 (23)	51 (20)	
Heterosexual and intravenous drug use (%)	45 (14)	35 (13)	
Other/unknown	12 (4)	13 (5)	
Median T CD4+ cell count − cells/mm^3^ (range)	427 (38–1612)	321 (5–1777)	**0.001**
Detectable HIV viral load (>40 copies/ml)*	98 (32)	125 (48)	**<0.0001**

MSM: men who have sex with men.

* Two patients in the HCV non-referred group did not have available HIV viral loads.

**Table 2:** Common clinical characteristics that may influence medical providers' decision of referral HIV-infected patients for hepatitis C treatment consideration.

Clinical characteristic	Referred n = 303 (%)	Non-referred n = 259 (%)	*P* value
Not engaged in care	31 (10)	95 (37)	**<0.0001**
Unstable housing	22 (7)	39 (15)	**0.003**
Most recent CD4 cell count ≤200 cells/mm^3^	51 (17)	70 (27)	**0.003**
Ongoing neuropsychiatric disorders	143 (47)	62 (24)	**<0.0001**
Ongoing drug and/or alcohol abuse	118 (39)	75 (29)	**0.013**
Decompensated cirrhosis	34 (11)	21 (8)	0.22
Active opportunistic infections	5 (2)	9 (4)	0.17
Platelets ≤50,000/mm^3^ *	11 (4)	6 (2)	0.37
Anemia ≤10 g/dL*	11 (4)	10 (4)	0.89
Malignancy other than hepatocellular carcinoma	6 (2)	15 (6)	**0.02**
Cardiovascular disease	16 (5)	13 (5)	0.89
Chronic kidney disease 3b (<45 mL/min/1.73m^2^)	10 (3)	13 (5)	0.31
Other medical comorbidity that takes clinical care priority	26 (9)	14 (5)	0.14

*Two patients in the HCV non-referred group did not have available hemoglobin and platelet values.

Using bivariate categorical analysis, predictors for not-being referred for HCV therapy evaluation included lack of engagement in care [odds ratio (OR) 5.08, 95% confidence interval (CI) 3.24–6.97], having unstable housing (OR: 2.26, 95% CI 1.30-3.93), AIDS (OR: 1.83, 95% CI 1.22-2.75), a detectable HIV viral load (OR: 1.98, 95% CI 1.36-2.75), an active malignancy (OR 3.47, 95% CI 1.35-8.92) and being non-white (OR: 1.67, 1.17-2.38). Ongoing psychiatric disorder and drug/alcohol abuse were negatively associated with non-referral for HCV therapy (OR: 0.35, 95% CI 0.26-0.63 and OR: 0.37, 95% CI, 0.24-0.56, respectively). Multiple logistic regression analysis adjusting for all aforementioned significant variables showed that the strongest predictor for non-referral to HCV therapy was being not-engaged in care (OR: 4.42, 95% CI 2.69-7.28), Table 3. Data fidelity abstraction was verified by a random audit of 175 charts with particular attention to classification of ongoing barriers to care. We found 97.74% agreement between observers in overall diagnostic criteria with a corresponding coefficient kappa  = 0.81 (95 CI: 0.62-0.99).

**Table 3:** Predictors for non-being referred for HCV therapy consideration (n = 562) in unadjusted and adjusted analyses.

Covariate	Unadjusted OR (95% CI)	*P* value	Adjusted OR (95% CI)	*P* value
Not engage in care	5.08 (3.24–6.97)	<0.000001	4.42 (2.69–7.28)	<0.000001
Having detectable HIV load* (>40 copies/mL)	1.98 (1.36–2.75)	0.0001	1.88 (1.26–2.81)	0.002
Unstable housing	2.26 (1.30–3.93)	0.004	1.97 (1.03–3.79)	0.041
Having an active malignancy	3.47 (1.35–8.92)	0.01	2.78 (0.96–8.03)	0.058
AIDS (CD4 cell count <200/mm3)	1.83 (1.22–2.75)	0.004	1.68 (1.05–2.69)	0.030
Non-white race	1.67 (1.17–2.38)	0.005	1.59 (1.06–2.40)	0.025
HIV risk factors (reference intravenous drug use)				
Men having sex with men	1.96 (1.34–2.87)	0.0006	1.96 (1.27–3.02)	0.0023
Other	2.51(1.54–4.07)	0.0002	1.64 (0.95–2.82)	0.075
Ongoing neuropsychiatry condition	0.35 (0.26–0.63)	<0.000001	0.37 (0.24–0.56)	<0.000001
Ongoing drug and/or alcohol abuse	0.64 (0.45–0.91)	0.013	0.52 (0.34 –0.80)	0.0027

*Two patients in the HCV non-referred group without available HIV viral loads were removed from final analyses.

**Figure 1:**
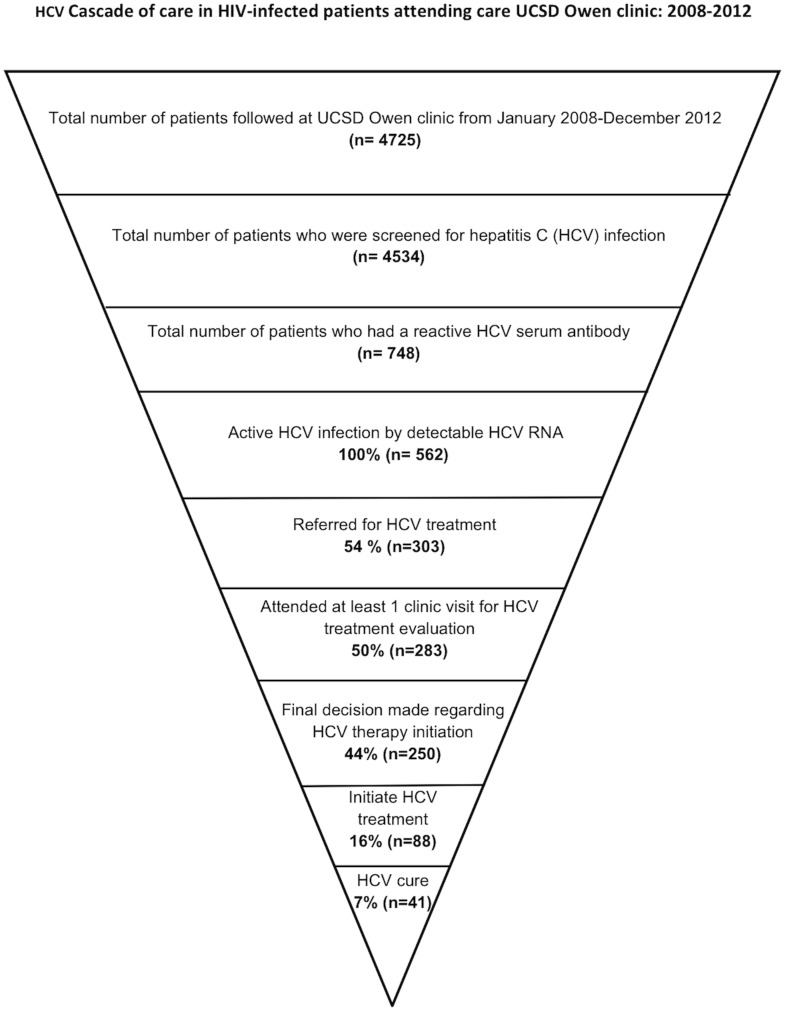
The figure depicts the hepatitis C cascade of care among HIV-infected patients who attended the University of California, San Diego Owen Clinic between 1 January 2008 and 31 December 2012.

From the 303 patients referred for HCV therapy, 250 patients completed staging for HCV treatment and 88 initiated HCV therapy. Of those, 22 were treated with pegylated interferon, ribavirin and telaprevir. Ultimately, 41 patients were cured of HCV. Figure 1 depicts the overall HCV cascade of care among patients enrolled in HIV care at UCSD Owen Clinic with known diagnosis of active HCV infection.

The main reason for not initiating HCV therapy in the 195 patients who completed HCV staging was the presence of ongoing barriers to care [78 of 195 (40%)]. Other reasons included: patient choice to wait for interferon-sparing regimens [45 of 195 (23%)]; history of advanced liver disease [34 of 195 (18%)]; prior HCV treatment failure with low chances of eradication with available HCV therapies [16 of 195 (8%)], undetectable HCV viral load due to spontaneous clearance [16 of 195 (8%)], and severe medical comorbidities that precluded use of interferon-based therapy [6 of 195 (3%)]. Despite having an inclusive protocol for the treatment of vulnerable patients with ongoing barriers to care, 78 patients could not start HCV therapy. The barriers to care in our referred for but not treated co-infected patients included: ongoing drug/alcohol abuse associated with lost to follow-up (32 of 78 (41%)]; ongoing drug/alcohol abuse associated with unwillingness to participate in the clinic protocol to qualify for HCV therapy [18 of 78 (23%); uncontrolled neuropsychiatric disorder (15 of 78 (19%)], and uncontrolled HIV disease in the context of ongoing drug or alcohol abuse [13 of 78 (17%)]. In the era of interferon-free HCV treatment, if we exclude from the group of 195 non-initiators the 16 patients who had subsequent undetectable HCV viral load, most patients who completed HCV staging and who did not initiate HCV therapy [101 of 179 (56%)] would benefit from these new HCV interferon-free treatment options; however, in a significant proportion of patients [78 of 179 (44%)], interferon-free combinations may not be enough to cure their HCV unless ongoing barriers to care are effectively addressed and managed.

## Discussion

Our findings lead to the following conclusions:(1) most HIV-infected patients linked to care were screened for HCV; (2) approximately half of HIV-infected patients diagnosed with HCV coinfection were not referred for HCV therapy; (3) the most common reason for not initiating HCV therapy was the presence of ongoing barriers to care that compromised their engagement in care.

The study results reflect extant findings regarding health disparities in access to care in the United States [13]. Non-white patients with lack of engagement in care, unstable housing and uncontrolled HIV-infection were referred less frequently for HCV therapy. Patients with a history of IDU were more likely to be referred for HCV therapy than MSM without IDU history. It is unknown to what degree patients themselves did not want to be referred because of stigma to HCV in the MSM not IUD community [14]. This creates an opportunity for increased awareness among HIV medical providers and patients of the rising prevalence of HCV among HIV-infected MSM in the absence of IDU [15]. About 50% of HIV coinfected patients with ongoing HCV infection were not referred for HCV therapy. We found that having interferon contraindications was not a factor for lack of HCV referral, neither was patients' unwillingness to receive HCV interferon-base therapy from detailed review of clinic notes. This highlights that, although we will have interferon free regimens with high cure rates in the immediate future, if these barriers to referral are not addressed, a large proportion of patients will never be linked to receive HCV treatment [16], [17].

It was unexpected that ongoing substance abuse or neuropsychiatric disorders were positive predictors of HCV treatment referral. Ascertainment bias is the most likely explanation for this finding [18]. Patients who were not referred for HCV therapy were less engaged in care, and the reliability of self-reported ongoing drug or alcohol abuse in this group of patients is uncertain. Similarly, there were more systematic evaluations and documentation of alcohol, drug abuse and neuropsychiatric disorders in the electronic medical records of patients who were referred for HCV therapy.

This study found that the most common factor associated with non-initiation of HCV therapy was attributed to the combination of drug, alcohol abuse or neuropsychiatric disorders that also affect patient's engagement in care; however, among patients who were referred for HCV therapy, the study findings suggest that even in a real-world challenging population, successful HCV therapy can be accomplished. While the numbers are small, almost 50% (41/88) of those starting therapy attained a sustained viral response. Response rates are only expected to increase as interferon-free therapies that are highly efficacious in shorter time are approved [19]. Emphasis needs to be placed on implementing models of care with integrated multidisciplinary treatment services for HIV and HCV, including treatment of alcohol and substance use, without stigma and discrimination [20]–[22]. This strategy may increase the number of HIV coinfected patients linked to and retained in care who could also initiate and complete HCV curative therapy [23].

Our study has several limitations. First, there was no information on insurance status and income level that in most health systems influences referral to HCV therapy [24]. Our HCV coinfection clinic operates within the main HIV clinic and no additional insurance coverage is required, allowing our patients universal access to HCV therapy evaluation. Second, it could be argued that our definition of engagement in care is not the gold standard as there are other measures such as missed visits, gaps in care and visit constancy that could provide us a more comprehensive description of patient's level of engagement in care [25]. We, however, chose the HRSA HIV/AIDS Bureau performance measure for retention in HIV care that aligns with the updated HIV Medicine Association primary HIV care guidelines that highlight the concept of adherence to clinical care [2]. Third, restricting the analysis of reasons for not initiating HCV therapy to only those patients who finish HCV clinical staging could be criticized. In fact, 53 of 303 (17%) patients referred for HCV therapy either never showed up for their HCV appointment (n = 20) or did not return after their first HCV appointment (n = 33). We cannot rule out that other unmeasured factors, such as travel distance to our clinic and low HCV health literacy, may have negatively impacted HCV therapy initiation [26], [27]; however, this limitation is balanced by the strength that most patients who completed HCV clinical staging underwent a detailed characterization of common medical, social and logistical barriers that influence HCV treatment initiation in clinical practice. Finally, as with other single site studies, our results may not be generalizable due to many factors such as different funding mechanisms of health care systems in different geographic areas; however the percentage of HIV coinfected patients who ultimately achieved HCV sustained viral response in this study mirrors recent reports in other clinical cohorts in the United States and Europe [28], [29].

In conclusion, despite improvements in HCV therapy the benefits will not be realized unless effective measures for dealing with barriers to care are identified and addressed.

## Supporting Information

File S1 Contains the following supporting information files: **Table S1.** Describes common medical conditions in the studied population where use of pegylated interferon and/or ribavirin could be contraindicated. **Table S2**. Describes medical comorbidities present in the group of HIV-infected patients referred or not for HCV therapy that could influence decision for hepatitis C referral (Table S2 in File S1).
(DOCX)
