# Supplementary material for: The Hepatitis C Cascade of Care among HIV Infected Patients: A Call to Address Ongoing Barriers to Care
Source: PLoS One. 2014 Jul 18;9(7):e102883. doi: 10.1371/journal.pone.0102883 (PMC4103859; doi:10.1371/journal.pone.0102883)
Supplement: File S1 — Contains the following supporting information files: Table S1. Describes common medical conditions in the studied population where use of pegylated interferon and/or ribavirin could be contraindicated. Table S2. Describes medical comorbidities present in the group of HIV-infected patients referred or not for HCV therapy that could influence decision for hepatitis C referral (Table S2 in File S1). (DOCX) [file pone.0102883.s001.docx]

Table S1. Common medical conditions where use of pegylated interferon and/or ribavirin could be contraindicated

| **Pulmonary conditions** |
| --- |
| Chronic obstructive pulmonary disease Stage III or higher FEV1/FVC<49-30% |
| Pulmonary Hypertension class III or higher according to the World Health Organization functional assessment classification |
| Restrictive lung disease with moderate functional impairment (Symptoms with less than ordinary activity. Marked limitation of activity) |
| Severe obstructive sleep apnea |
| **Hematologic conditions** |
| Pancytopenia |
| Thrombotic thrombocytopenic purpura |
| History of leukopenia depending on growth-stimulating factors therapy |
| **Dermatologic conditions** |
| History of difficult to control psoriasis |
| Chronic eczema extending to 3 or more locations in body |
| **Neurologic conditions** |
| Dementia |
| Difficult to control seizures : at least one episode every three months despite treatment with multiple medications |
| Dementia |
| Traumatic encephalopathy |
| Recent (within 3 months) cerebrovascular accident |
| Progressive multifocal leukoencephalopathy |
| Severe dyskinesia |
| **Gastrointestinal conditions** |
| Recent pancreatitis (within 3 months) |
| Acute cholecystitis |
| **Renal conditions** |
| Uncontrolled nephrotic syndrome |
| Ongoing interstitial nephritis |
| **Rheumatologic conditions** |
| Active or moderately symptomatic rheumatoid arthritis |
| Moderate symptomatic or higher polymyalgia rheumatica |
| Endocrine conditions |
| Active or difficult to control hyperthyroidism despite therapy |
| **Pregnancy** |
| **Active infections or recent solid organ transplants such as:** |
| Active spine tuberculosis infection |
| Renal transplant |
| Endocarditis |

Table S2. Description of medical comorbidities present in the group of HIV-infected patients referred or not for HCV therapy that could influence decision for hepatitis C referral.

| Clinical condition* | Non-referred group  (Total No patients =26) | Non-referred group  (Total No patients =14) |
| --- | --- | --- |
| Pulmonary hypertension moderate | 3 | 1 |
| COPD | 5 | 2 |
| Restrictive lung disease | 1 | 0 |
| Severe obstructive sleep apnea | 1 |  |
| Thrombotic thrombocytopenic purpura | 1 | 1 |
| Difficult to control seizure disorder | 2 | 2 |
| Traumatic encephalopathy with severe residual cognitive impairment | 1 | 0 |
| Severe tardive dyskinesia | 1 | 0 |
| Progressive multifocal leukoencephalopathy | 0 | 1 |
| Dementia | 0 | 3 |
| Recent cerebrovascular accident | 1 | 1 |
| Severe psoriasis | 2 | 0 |
| Moderate rheumatoid arthritis | 1 | 0 |
| Polymyalgia rheumatica | 1 | 0 |
| Disseminated tuberculosis-active | 2 | 1 |
| Endocarditis -active | 1 | 0 |
| Interstitial nephritis | 1 | 0 |
| Nephrotic syndrome | 1 | 0 |
| Hyperthyroidism- active | 1 | 0 |
| Renal transplant | 1 | 0 |
| Cholelithiasis | 1 | 1 |
| Recent pancreatitis | 0 | 1 |
| Pregnancy | 0 | 1 |

*Some patients may had more than 1 clinical condition
